# Dynamic tracking of manganese uptake in mouse hearts by rapid multi-slice T_1 _mapping

**DOI:** 10.1186/1532-429X-16-S1-W35

**Published:** 2014-01-16

**Authors:** Kai Jiang, Xin Yu

**Affiliations:** 1 Biomedical Engineering, Case Western Reserve University, Cleveland, Ohio, USA

## Background

Manganese (Mn^2+^)-enhanced MRI (MEMRI) has the potential for in vivo assessment of the voltage-gated L-type Ca^2+ ^channel activity. Quantitative assessment of Mn^2+ ^uptake via Ca^2+ ^channels requires fast and accurate T_1 _mapping. In the current study, a multi-slice saturation recovery Look-Locker (MSRLL) method was developed for T_1 _mapping of the whole mouse heart in < 3 min.

## Methods

### MSRLL Sequence

**Figure 1:**
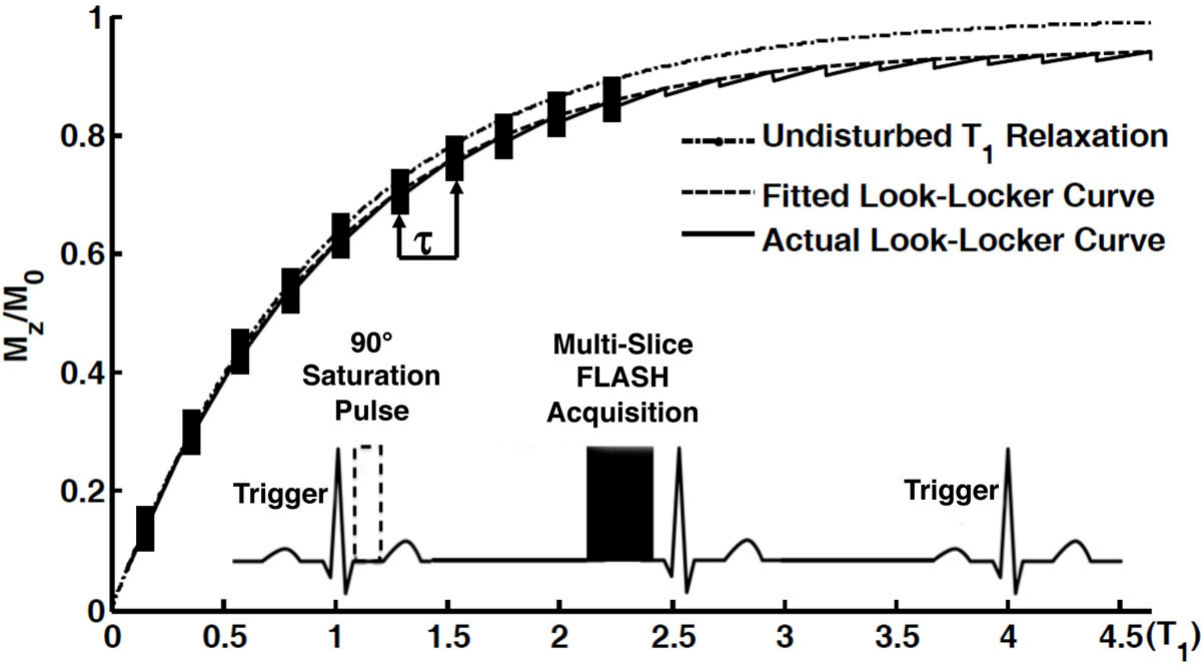
**MSRLL pulse sequence**. After 90° non-slice selective saturation pulses, 10 sequential multi-slice FLASH acquisitions separated by an interval τ were applied. Each block on the relaxation curve represents a multi-slice acquisition module.

A schematic diagram of the MSRLL pulse sequence is shown in Figure 1. An ECG-triggered saturation module was applied at the beginning of each phase-encoding step, followed by the acquisition of k-space lines along the magnetization recovery curve in multiple slices. ECG-triggered image acquisition was performed at late diastole.

### Phantom Study

All MRI studies were performed on a horizontal 7.0T Bruker scanner with a 35 mm volume coil. The MSRLL method was first validated in vitro using a multi-compartment phantom with MnCl_2 _solution ranging from 30 μM to 1000 μM. T_1 _maps of 5 slices were compared with those acquired with a previously validated single-slice method (SRLL). Imaging parameters were: flip angle, 10°; TE, 1.9 msec; slice thickness, 1 mm; number of average, 1; field of view, 3 × 3 cm^2^; matrix size, 128 × 64.

### In Vivo Study

In vivo MEMRI studies were performed in 3-month-old FVB mice (n = 19). T_1 _maps of three adjacent short-axis slices at mid-ventricular levels were acquired with the same imaging parameters as those used in vitro. Continuous T_1 _mapping was performed during the 30 min of MnCl_2 _infusion through tail vein (0.2 mL/hr) and the 15 minutes washout. To investigate the Mn^2+^-induced relaxivity changes, two different MnCl_2 _solutions at 126 mM (n = 9) and 63 mM (n = 10) were used. Validation study was performed either at baseline (n = 10) or post-contrast (n = 10).

## Results

**Figure 2:**
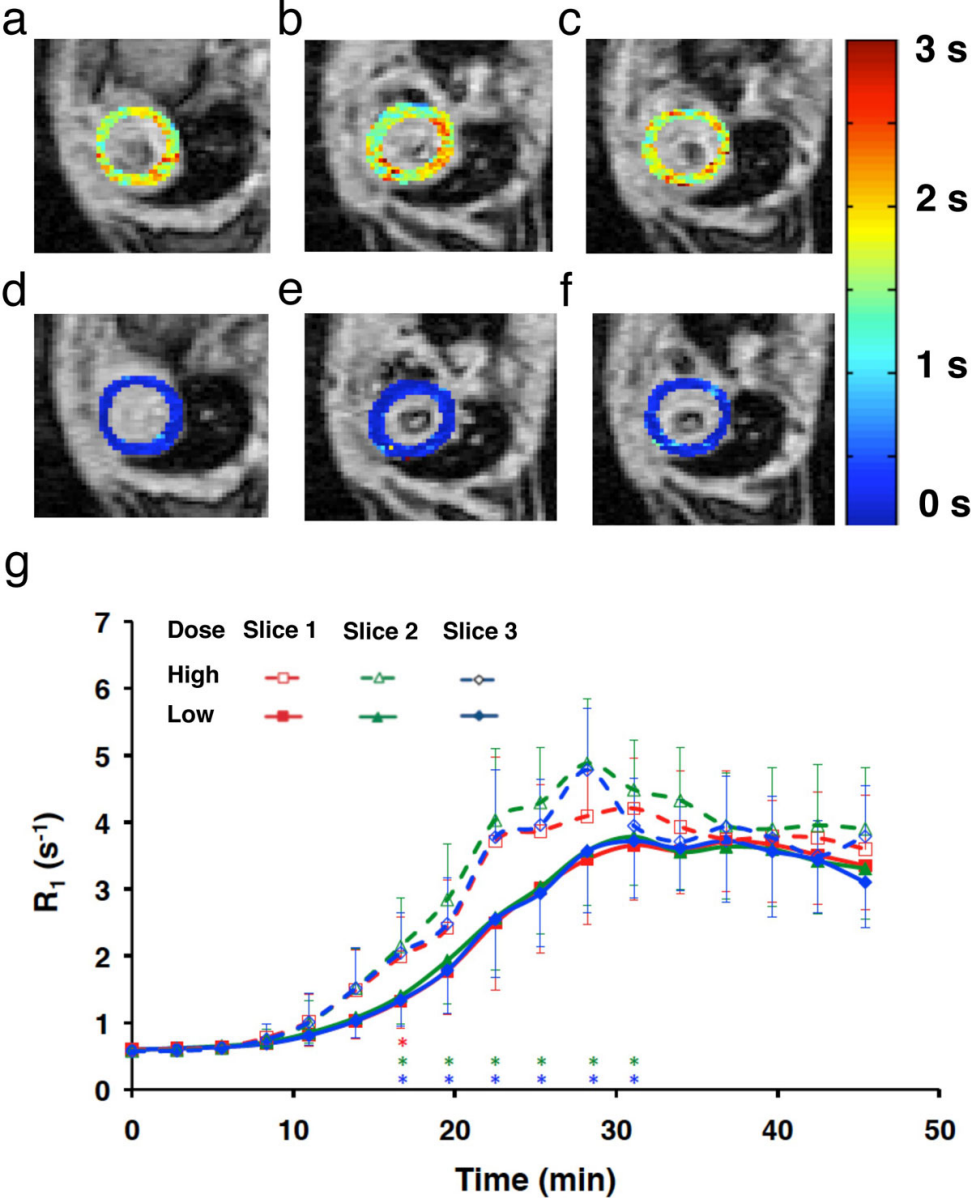
**R_1 _changes in dynamic MEMRI study**. a-c. Pre-contrast T_1 _maps of the three slices. d-f. Post-contrast T_1 _maps of the three slices. g. Time courses of R_1 _changes.

In vitro studies showed strong agreement between MSRLL and SRLL. Average imaging time in vivo was 140~166 s. Shown in Figure 2 are representative T_1 _maps acquired at baseline (Figure 2a-c) and after Mn^2+ ^infusion (Figure 2d-f). All three slices showed significant reduction in T_1 _after Mn^2+ ^infusion. The time courses of the R_1 _changes for all three slices are presented in Figure 2g. In general, higher Mn^2+ ^dose induced larger increase in R_1 _during Mn^2+ ^infusion.

## Conclusions

An ECG-triggered, multi-slice saturation-recovery Look-Locker method was developed for fast and complete cardiac T_1 _mapping in mice. Validity and utility of this method was well demonstrated in the phantom and in vivo two-dose MEMRI studies.

## Funding

This study was supported by NIH Grants HL73315 and HL86935 (Yu).

